# Poster Session I - A26 FUNCTIONAL INTERACTION OF SIRT1 AND HNF4A IN INTESTINAL EPITHELIAL HOMEOSTASIS

**DOI:** 10.1093/jcag/gwaf042.026

**Published:** 2026-02-13

**Authors:** J A Herrera Pulido, C Jones, S Giraud, F Boudreau

**Affiliations:** Immunology and cell biology, Universite de Sherbrooke, Sherbrooke, QC, Canada; Immunology and cell biology, Universite de Sherbrooke, Sherbrooke, QC, Canada; Centre de Recherche en Cancérologie de Lyon, Lyon, Rhône, France; Immunology and cell biology, Universite de Sherbrooke, Sherbrooke, QC, Canada

## Abstract

**Background:**

The intestinal epithelial barrier is essential for homeostasis, relying on a cycle of cell proliferation, differentiation, and death. Disruption of this process is linked to inflammatory bowel diseases. HNF4A, a nuclear transcription factor, and SIRT1, a deacetylase, are key regulators of this barrier, with their loss being associated with IBD and colorectal cancer. In HCT116 CRC cells, we found a direct interaction between HNF4α2 and SIRT1, but no evidence of changes in acetylation of HNF4A levels was observed. The molecular mechanisms and physiological significance of this interaction in maintaining the epithelial barrier and in disease contexts are still unclear.

**Aims:**

To characterize the interaction between HNF4A2 and SIRT1 in intestinal epithelial cells at the biological and molecular level.

**Methods:**

We validated the interaction between HNF4A2 and SIRT1 using AlphaFold3, PyMOL 4.6.0, and ChimeraX 1.10.1, referencing UniProt crystal structures. Our findings were confirmed through a GST pull-down assay with GST-tagged HNF4A2 domains and a SUMO-tagged SIRT1 protein. To explore the biological significance, we recreated an epithelial barrier using Transwell cultures from human sigmoid colon organoids in differentiation medium. We analyzed the expression of HNF4A and SIRT1 and evaluated the effects of commensal bacterial infection. Additionally, we created two organoid lines using CRISPR-Cas9 to silence either SIRT1 or HNF4A, assessing their roles in epithelial barrier integrity.

**Results:**

In silico analyses revealed an interaction between HNF4A and the closed structure of SIRT1, which is mediated by the C domain of HNF4A. This interaction was confirmed through pull-down assays. These findings, along with preliminary results from MS, suggest that SIRT1 does not perform a deacetylation function on HNF4A. Instead, it could recruit other proteins that regulate the transcriptional activity of HNF4A. At the biological level, we found that intestinal epithelial cells express low levels of SIRT1 and P2 family isoforms of HNF4A when cultured in a differentiation medium. In contrast, the P1 family isoforms show high levels of expression. However, these proportions change in response to bacterial infection. These observations imply that the interaction between SIRT1 and HNF4A may play a role in regulating cell proliferation and differentiation at the epithelial level. Finally, when either protein is lost, we observe a disruption in the integrity of the epithelial barrier.

**Conclusions:**

The findings indicate that SIRT1 is essential for regulating HNF4A transcriptional activity in the intestinal epithelium, affecting cell proliferation and differentiation to maintain epithelial barrier integrity. Further research is needed to fully understand this interaction, which could lead to new strategies in designing therapies for intestinal epithelial disorders.

**Funding Agencies:**

CAG, CIHR

